# Segmenting the Semi-Conductive Shielding Layer of Cable Slice Images Using the Convolutional Neural Network

**DOI:** 10.3390/polym12092085

**Published:** 2020-09-14

**Authors:** Wen Zhu, Fei Dong, Beiping Hou, Wesley Kenniard Takudzwa Gwatidzo, Le Zhou, Gang Li

**Affiliations:** School of Automation and Electrical Engineering, Zhejiang University of Science and Technology, Hangzhou 310023, China; joywenzhu@126.com (W.Z.); donffe@163.com (F.D.); wesleyzust@163.com (W.K.T.G.); zhoule@zust.edu.cn (L.Z.); ieligang@zjut.edu.cn (G.L.)

**Keywords:** semiconductive shielding layer, image segmentation, convolutional neural network

## Abstract

Being an important part of aerial insulated cable, the semiconductive shielding layer is made of a typical polymer material and can improve the cable transmission effects; the structural parameters will affect the cable quality directly. Then, the image processing of the semiconductive layer plays an essential role in the structural parameter measurements. However, the semiconductive layer images are often disturbed by the cutting marks, which affect the measurements seriously. In this paper, a novel method based on the convolutional neural network is proposed for image segmentation. In our proposed strategy, a deep fully convolutional network with a skip connection algorithm is defined as the main framework. The inception structure and residual connection are employed to fuse features extracted from the receptive fields with different sizes. Finally, an improved weighted loss function and refined algorithm are utilized for pixel classification. Experimental results show that our proposed algorithm achieves better performance than the current algorithms.

## 1. Introduction

The semiconductive shielding layer is an important part of the aerial insulated cable; it is made of polymer material to balance the electric field distribution and avoid partial discharge. In trying to improve the reliability of this material when used in cables above 10 kV, more accurate and intelligent methods for structural parameter measurement are required during the polymer manufacturing process.

Traditionally, a series of cursory measuring points are selected by the naked eye and then measured manually with some tools such as a vernier caliper. Although optical instruments such as microscopes and projectors have been introduced in recent years, it is still hard to find the real weak points in insulation and shielding layers manually. Manual measuring methods are low in efficiency, poor in repeatability, and cumbersome in operation, while the results are usually affected by human factors.

In recent years, methods based on machine vision have been developed to solve the problems in manual measurement mentioned above. Cui [1] compared several edge detection operators used for contour extraction of cable slices and found that the binary morphology operator yields the best performance in edge feature detection. Fan [2] proposed a system to obtain the insulation contours by means of total variation denoising and the binary morphology operator. Feng [3] adopted the spindle transformation and multi-scale gradient to improve the precision of edge location. An improved Sobel–Zernike moment positioning method was proposed by Xia [4] to enhance the speed of the sub-pixel location method, while Bian [5] proposed an improved sub-pixel interpolation algorithm for cable thickness measurement.

Although there exist works on cable structural parameter measurement, most of them just focus on the measurement of the insulation layer and sheath layer for conventional types of regular cables. While parameter measurement of the semiconductive shielding layers mainly relies on manual measurement combined with a projector, there are some shortcomings, and this cannot meet the increasing requirements of cable security.

The fast segmentation of the semiconductive shielding layer is a key step in the process of vision measurement, but it is hard to extract features by traditional image processing methods because the semiconductive layer image regions are often seriously disturbed by the cutting marks.

Convolutional neural networks (CNNs) have become effective methods applied in image classification and segmentation [6,7,8,9,10]. Fully convolutional networks (FCNs) remove the fully connected layer of the typical classification network and reconstruct the resolution of feature maps for image segmentation. FCN-based approaches are used to solve some specific problems in image segmentation [11,12,13,14,15].

In our research, an FCN-based algorithm is presented to acquire the image region of the semiconductive shielding layer automatically. After analyzing the characteristics of aerial insulated cable images, the inception structure and residual connection are utilized to calculate the details for network, an improved weighted loss is proposed to locate the outer border of the semiconductive shielding layer, and pixels similar to the foreground can also be found. During the segmentation process, there will be some mispredicted regions. Finally, a refinement step is proposed to remove the interference regions according to the prior knowledge and then ensure the unique region of the semiconductive shielding layer.

## 2. Image Analysis of Aerial Insulated Cable Slices

**Figure 1:**
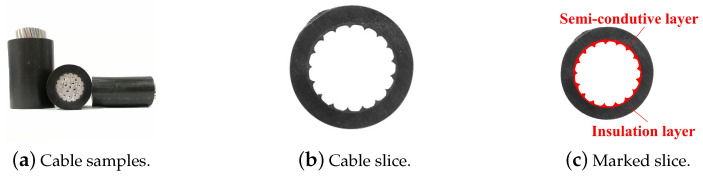
Cable samples and slices.

The first step in measuring the structural parameters of an aerial insulated cable is sampling. A cable slice is then cut from the sample. Some samples with a conductor (a) and a cable slice without a conductor (b,c) are shown in Figure 1.

The images of the aerial insulated cable slice, as shown in Figure 1b,c, consist of two parts, the insulation layer region and the semiconductive shielding layer region, as shown in Figure 1c.

As a whole, the structure of the insulated cable slice is circular, while the internal part is a sawtooth shape. In terms of spatial position, the semiconductive layer is located in the inner layer and closely adheres to the insulation layer.

**Figure 2:**
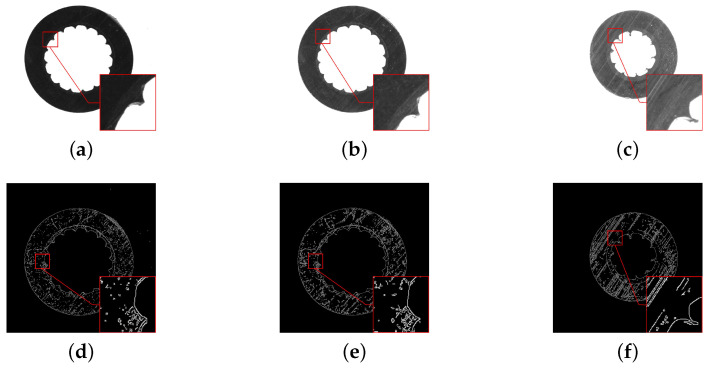
Collected cable slice images and their corresponding edge detection results: (**a**–**c**) show the different cable slices under different illumination, while (**d**–**f**) shows the corresponding edge images obtained by the Canny operator.

Three typical aerial insulated cable slices, which were taken under different illumination, are shown in Figure 2a–c. In each image, the typical region is marked by a red rectangle for analysis, and the selected regions are enlarged in the lower right corner. As is seen in Figure 2a,c, the colors of the two layers are so similar that it is difficult to distinguish their actual boundary. As is seen in Figure 2b,c, the two layers fit very closely, and it is difficult to distinguish their boundary. According to the cutting process, the cutter will leave parallel stripes on the surface of the cable slice when a cable slice is made. As seen from Figure 2b,c, the enlarged image regions display the evident parallel stripes, which cover the whole image regions, and change the original texture structure of the slice images.

The regular image segmentation methods include threshold segmentation, edge detection, etc. The Canny operator is a widely used edge detection algorithm with good performance. The results of the Canny operator for edge extraction in Figure 2a–c are shown in Figure 2d–f. It is simple to detect the inner and outer edges, but it is hard to obtain the boundary between the two layers completely, because the gray value of these parallel stripes is close to that of the semiconductive shielding layer.

At the same time, these images are illuminated differently. When the light is dim, as shown in Figure 2a, both the cutting marks and the semiconductor shielding layer are difficult to recognize. When the light is bright, both the cutting marks and the semiconductor shielding layer are clear. When the brightness is medium, as shown in Figure 2b, there are still many cutting marks, and the segmentation task cannot be completed, as shown in Figure 2e.

From the above, due to the similar color of the two layers and the cutting marks, it is difficult to locate the region of the semiconductor shielding layer. Then, the FCN-based method is proposed to solve the problems above.

## 3. Preliminaries

### 3.1. Convolution Block

A convolution block usually consists of convolutions, batch normalization (BN) [16], and the activation function. The filters of convolutions slide over images to extract features. The BN algorithm [16] is commonly followed by convolution to accelerate the convergence of the network and prevent the network from overfitting. The activation function introduces non-linear decision boundaries to the network, and the rectified linear unit (ReLU) is often employed in deep learning applications, as it can alleviate the vanishing gradient problem and is considerably faster than the alternatives.

### 3.2. Inception Structure

**Figure 3:**
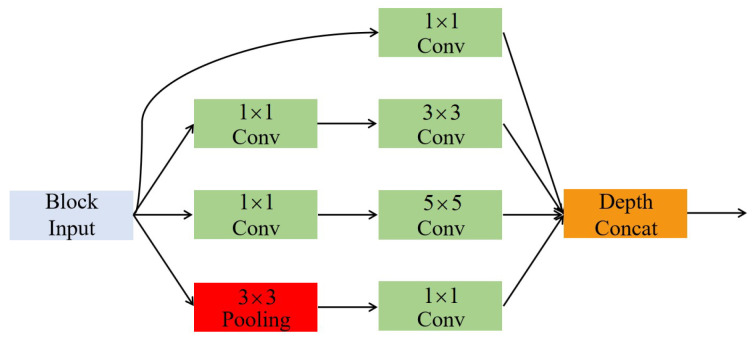
Inception structure.

The inception structure [6] stacks convolutions with the kernel sizes of 1 × 1, 3 × 3, and 5 × 5 and pools together, as shown in Figure 3. According to Szegedy et al. [6], it can increase the width of the network and improve the adaptability for scales. Szegedy [7] also proved that the parameters can be reduced by repeated application of the 3 × 3 convolution instead of the 5 × 5 convolution.

### 3.3. Residual Connection

**Figure 4:**
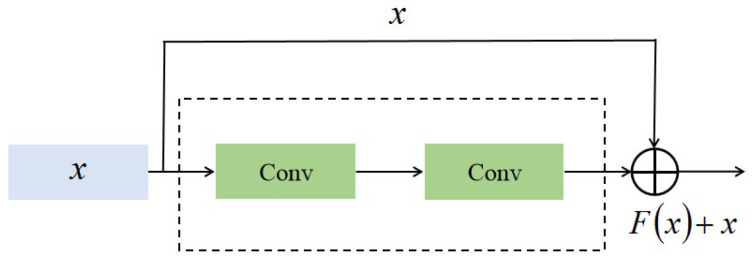
Convolutions with a residual connection.

In the residual connection algorithm [17], shortcuts are used as the identity mapping to propagate the gradients of the network. A residual unit that introduces shortcuts between convolutions is shown in Figure 4, and it adds an identity mapping that converts the original output to F(x)+x, where *x* denotes the input features.

### 3.4. U-Shaped Structure

The U-shaped structure [18] consists of a contracting path and an expansive path and is beneficial to detail the extraction and training of small datasets. The standard UNet [18] structure has five stages, which samples the images down to 1/16 and then samples up stage-by-stage for pixel-level prediction. The max-pooling layer is used to downsample the feature maps, while deconvolution is used to restore the resolution. The skip connection is used to fuse more low-level features in each stage.

## 4. The Proposed Method

### 4.1. Improved Network Architecture

**Figure 5:**
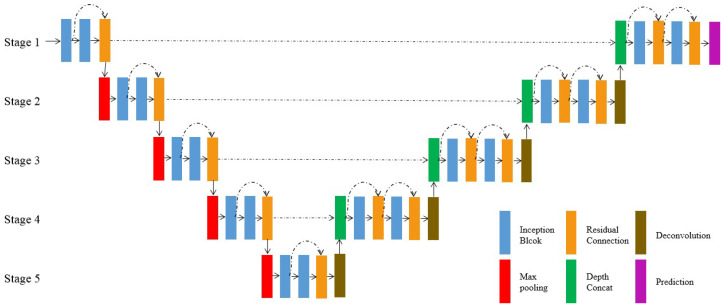
The block diagram of the proposed segmentation network.

The proposed network architecture is illustrated in Figure 5. It is designed on the basis of the U-shaped architecture [18], which consists of a contracting path and an expansive path. There are five stages in our architecture, and the information is passed from the contracting path to the expansion path at the corresponding stage through skip connections.

The contracting path gets the input images and outputs the feature maps of high-level semantics. Blocks with five stages and their feature map sizes are shown in Table A1. The first inception block extracts 32 feature maps from the input image, and then, another inception block follows. The resolution of the feature map is gradually reduced by using max-pooling stage-by-stage, while the channels of the feature map are doubled via the inception block following the max-pooling until it reaches 512.

**Figure 6:**
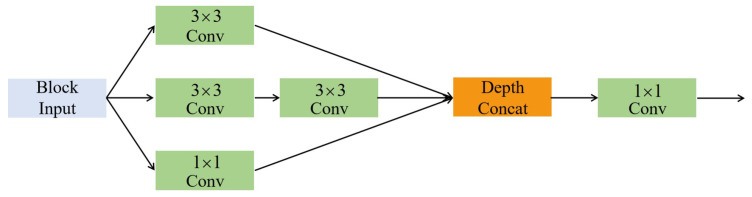
Inception block with three branches.

The residual connection is added to fuse the features extracted by the previous two blocks. More specifically, the proposed inception block is shown in Figure 6. It consists of three parallel branches with different sensory fields, and those branches are followed by a 1 × 1 convolution block to extract hybrid features. The first branch only contains one 1 × 1 convolution block, the second one 3 × 3 convolution block, and the rest the repeated application of two 3 × 3 convolution blocks instead of a 5 × 5 convolution block. These convolutions all use a stride of 1 × 1 with padding. The convolution block of the first inception block at each stage contains convolution, BN, and ReLU, while the second is output without ReLU. The max-pooling works with 2 × 2 kernels and a stride of 2 × 2, while the feature maps are sampled down to 1/16 of the original image after processing in five stages.

In the expansive path, the feature maps with high-level semantics and low resolution are up-sampled until they are restored to the size of the original image. The restored features from the last stage are concatenated with the features from the contracting path at the corresponding stage. These skip connections let low-level information be passed to higher levels directly and obtain richer features. The features at different scales are captured by the repeated application of the inception block at each stage, and residual connections are used to make the gradient spread efficiently. Then, the network can learn to build a more precise output based on the information. Starting with deconvolution at Stage 4, blocks and their feature map sizes are shown in Table A2. Each deconvolution doubles the resolution of the feature maps from the last stage and halves the number of channels; hence, its spatial dimension is consistent with the feature maps transferred by the skip connection. After the processing of an inception block, the information is fused, and the number of channels is halved. The information is further consolidated in the next inception block by the residual connection. Finally, the network fuses information and outputs a pixel-level prediction.

### 4.2. Improved Loss Function

The binary cross-entropy loss (BCE) is commonly used in binary classification and is defined as follows [19]:

(1) $$BCE\left(p,y\right)=\left\{\begin{array}{cc} -log(p), & \mathrm{if}\;y\;\mathrm{is}\;1\\ -log(1-p), & \mathrm{if}\;y\;\mathrm{is}\;0 \end{array}\right.$$

where y∈[0,1] is the pixel label, the foreground (y=1) and background (y=0). p∈[0,1] is the probability of the foreground estimated by the model and is computed by a sigmoid function over the output feature map. $p_{t}$ is defined to rewrite the BCE as $BCE\left(p,y\right)=BCE\left(p_{t}\right)=-log\left(p_{t}\right)$.

**Figure 7:**
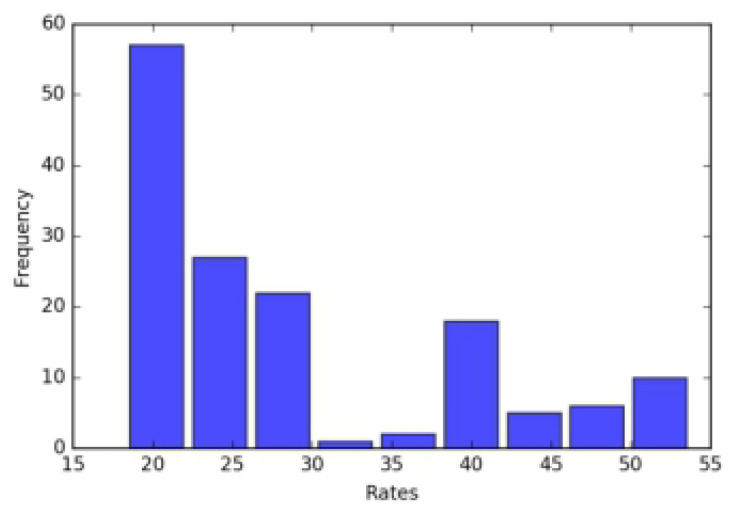
The histogram of the ratio between the pixel numbers of the background and foreground in the training data.

In the task of semiconductive shielding layer segmentation, pixels in the image are divided into the foreground and background, as shown in Figure 7, the background pixels in the image are much greater than the foreground pixels, which leads to the imbalance between the classes.

**Figure 8:**
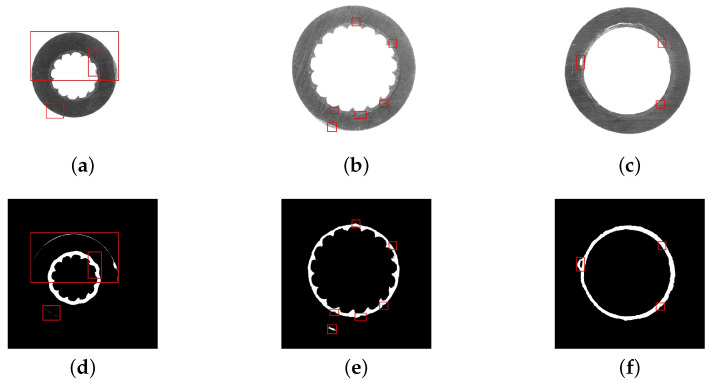
The coarse prediction of the network influenced by the pixels that are hard to classify. (**a**–**c**) are the source images and (**d**–**f**) the network prediction.

Furthermore, a basic UNet model is established, and the segmentation results are shown in Figure 8. The red rectangles mark the regions in the original images and prediction images where the prediction is obviously wrong. As seen from Figure 8a,d, the model mistook the outer part of the insulation layer for the semiconductor shielding layer. As seen from Figure 8b,e, the boundaries of the two layers are difficult to separate properly. Figure 8c,f shows that the model misidentifies parts of the insulation layer near the boundary as the semiconductor shielding layer. The outer edge of the insulation layer is similar to the semiconductive shielding layer in shape and color, then the network is prone to predicting it as the foreground.

The mentioned factors interfere with the segmentation results greatly. To solve the problem of class imbalance, a typical method is to introduce a weight factor into the loss function [18,19]. Ronneberger [18] used a weight map forcing the model to pay more attention to the border, and Lin [19] added a modulating factor making the model focus on the hard examples.

**Figure 9:**
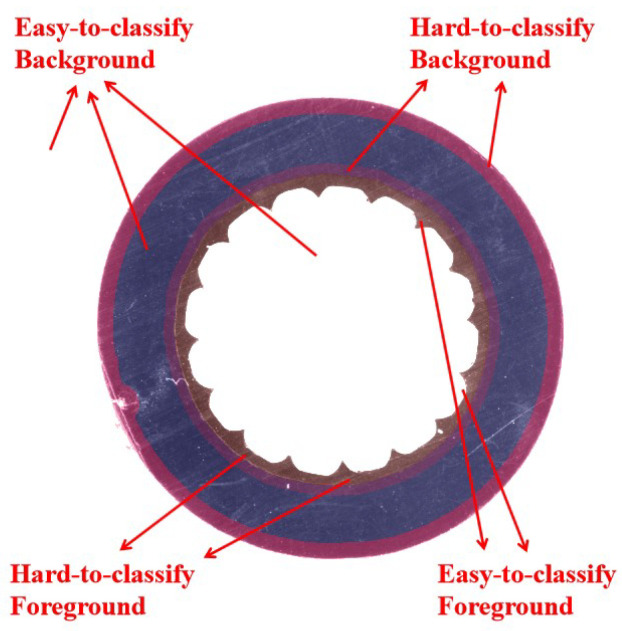
Segmentation difficulty of different regions.

As shown in Figure 9, the background outside the cable slice can be identified most easily, while the pixels on the boundary between the two layers are the hardest part to identify and is challengeable. Inspired by existing methods, a weighted loss is proposed to force the network to pay more attention to hard examples:

(2) $$WBCE\left(p,y\right)=\sum_{x\in \Omega }-\omega \left(x\right)log\left(p_{t}\right)$$

where *x* is the pixel position on the output feature map and ω is the weight map that we introduced to solve the problems mentioned above. The weight map contains three components and is pre-computed for each training datum:

(3) $$\omega \left(x\right)=k_{1}\omega_{c}\left(x\right)+k_{2}\omega_{p}\left(x\right)+k_{3}\omega_{h}$$

where $\omega_{c}$ is a class balance weight map, $\omega_{p}$ is a position weight map, and $\omega_{h}$ is a hard example penalty weight map. $k_{i}\in N\left(i=1,2,3\right)$ are parameters to adjust the proportion of the corresponding terms in ω. $\omega_{p}$ and $\omega_{h}$ are computed as:

(4) $$\omega_{p}\left(x\right)=\left\{\begin{array}{cc} exp(\frac{d_{p}\left(x\right)}{2\sigma_{1}}), & \mathrm{if}\;d_{p}\left(x\right)\leq 1\\ exp(-\frac{d_{p}\left(x\right)}{2\sigma_{2}}), & \mathrm{if}\;d_{p}\left(x\right)>1 \end{array}\right.$$

(5) $$d_{p}\left(x\right)=d_{i}\left(x\right)d_{o}\left(x\right)$$

(6) $$\omega_{h}\left(x\right)=exp\left(-\frac{d_{p}^{2}\left(x\right)}{2\sigma_{3}}\right)$$

where $d_{i}$ and $d_{o}$ are the minimum distances between the inner contour and outer contour of the semiconductive shielding layer at pixel position *x*, respectively. If the pixel is out of the contour, the distance is computed as a negative number; otherwise, the distance is positive. The position information of pixel *x* can be obtained according to the symbol and value of the distance. $\omega_{p}$ is computed according the position of the pixel. $d_{h}$ is the minimum distance between position *x* and the outer contour of the insulation layer. $\sigma_{i}\in N\;\left(i=1,2,3\right)$ are constants for the calculation of the effective distance.

**Figure 10:**
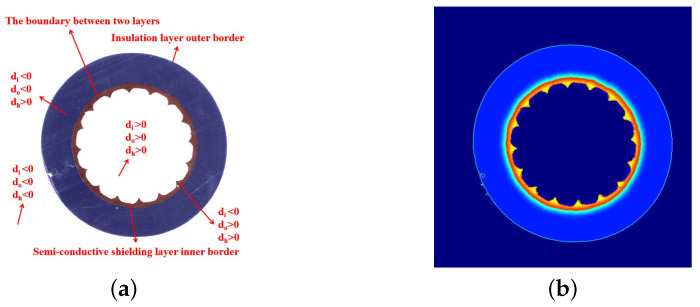
Weight map and its symbols in different regions. (**a**) Symbols of the distance between the pixels and the borders at different locations. (**b**) Pre-computed weight map.

As shown in Figure 10, *d* has different signs in different regions, and the weight map is calculated according to its values. The weight map is shown in Figure 10.

### 4.3. Prediction Refinement

In this section, the edge of the insulation layer is very similar to that of the semiconductive shielding layer in terms of geometry and texture.

**Figure 11:**
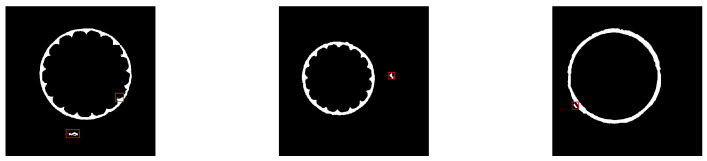
Examples of segmentation results with marked misclassified regions.

As shown in Figure 11, some isolated regions marked with red rectangles are predicted as the foreground, because the network is prone to classify the pixels of the outer edge of the insulation layer into the semiconductive layer. However, there is only one continuous semiconductive layer in one slice image. Thus, an approach based on the morphological properties of the target region is designed to remove the noise areas. Firstly, the network prediction is processed with an appropriate threshold value $t_{o}\in \left[0,1\right]$ according to function $T(p_{t})$:

(7) $$T\left(p_{t}\right)=\left\{\begin{array}{cc} 255, & \mathrm{if}\;p_{t}\geq t_{o}\\ 0, & \mathrm{if}\;p_{t}<t_{o} \end{array}\right.$$

Then, all the connected domains are found, and the aspect ratio of each region is calculated by its minimum enclosing rectangle.

(8) $$AR=\frac{R_{h}}{R_{w}}$$

where AR denotes the aspect ratio. $R_{h}$ and $R_{h}$ represent the height and width of the minimum enclosing rectangle, respectively.

Finally, small noises are removed according to the area of each region, and then, the regions are selected according to the aspect ratio with a threshold $t_{ar}$.

## 5. Experiments

### 5.1. Implementation

**Figure 12:**
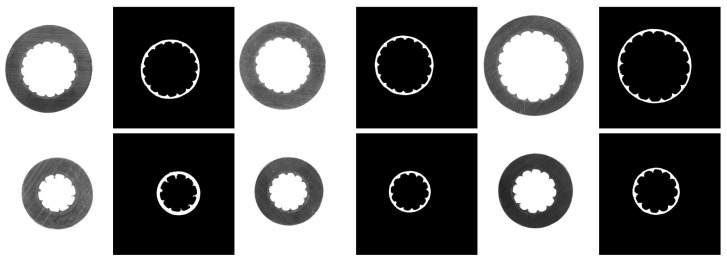
Images and the masks of the aerial insulated cable slice.

The proposed method is implemented with the TensorFlow framework, and the experimental environment included an Intel Core i5 3.4 GHz CPU, an NVIDIA GeForce GTX1060 GPU, and a 64 bit Windows operating system. The details of the experiment are given below:

- Dataset: A platform consisting of an industrial camera, a telecentric lens, and an auxiliary light source was set up to collect data, since it is difficult to collect the images with the semiconductive shielding layer by normal illumination. Two-hundred fifty-four images were collected from different aerial insulated cable sections under different lighting conditions, and then, the semiconductive shielding layers were manually labeled at the pixel level. The dataset was trimmed to a uniform size of 224 × 224 and was divided into a training set with 148 images, a validation set with 28 images, and a test set with 78 images. Cable slice images and their masks are shown in Figure 12. In the case of this task, the shift and rotation invariance, as well as the robustness to illumination variations were primarily considered. The content of this part is supplemented by two aspects.
- Evaluation metrics: Intersection over union (IoU), Dice coefficients, and pixel precision were used to evaluate the segmentation results. Let TP (true positive) be the number of pixels with the actual target predicted as the target, FP (false positive) be the number of pixels with the actual background predicted as the target, and FN (false negative) be the number of pixels with the actual target predicted as the background. Let KP be the number of pixels predicted as the target and KG be the number of pixels labeled as the target. The higher these metrics are, the better the model performs.
  (9) $$IoU=\frac{TP}{TP+FP+FN}$$
  (10) $$Dice=\frac{2TP}{KP+KG}$$
  (11) $$Precision=\frac{TP}{TP+FP}$$
- Parameter configuration: The weight maps were pre-calculated with the parameters of $\sigma_{1}=9$, $\sigma_{2}=25$, $\sigma_{3}=4$, $k_{1}=1$, and $k_{2}=k_{3}=5$. During the training phase, the sigmoid function was used to indicate the probability that each pixel is predicted to be the foreground, since there were only two classes in this task: foreground and background. The weighted binary cross-entropy loss function was optimized by gradient descent with a 0.001 initial learning rate. The network was trained with a mini-batch size of four for more than 100 epochs until the verified IoU no longer increased significantly. For the refinement step, we set $t_{o}=0.5$ and $t_{ar}=0.9$.

### 5.2. Results and Discussion

The proposed network structure has five stages, and the number of stages was adjusted to find the optimal numbers. As shown in Table 1, with the increase of the stages, the performance of the network improved gradually. However, the performance of the network can hardly be improved by adding stages after it reaches five stages.

In order to validate the effectiveness of the structure we improved, several submodels were built for comparison: S1, S2, S3. S1 is the standard UNet. In S2, the inception structure is introduced as the main building block of the network, and in S3, the residual connection is used on the basis of S2. Table 2 summarizes the performances of these models, and the results indicate that the performance can be enhanced by introducing the above two structures.

In order to validate the contribution of the prediction refinement steps, the predictions output by the models above were refined respectively. We found that there was an approximately 1% improvement in rough predictions, but little in the more robust models. However, this did work for some predictions. In this step, small regions that were incorrectly predicted would be filtered out without disturbing the target boundary. We tried to use fully connected CRFs [20] to refine as well, but that made the results worse, because of the cutting marks.

**Figure 13:**
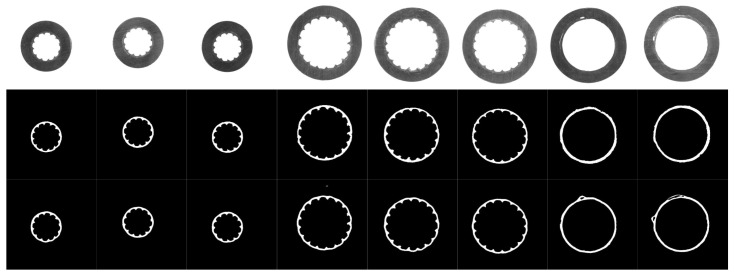
Segmentation results. The first row is the input images. The second row is the prediction of our method. The last is the results obtained by standard UNet.

The segmentation results are shown in Figure 13, and it shows a comparison of the segmentation results using our method with the standard UNet model. The first row in Figure 13 shows the cable slices at different sizes under different illumination; the second row displays the results of the proposed method; and the last row is the predicted results of standard UNet. It can be observed that the proposed method still has good segmentation results in the case of the severe interference of cutting marks, and it is robust to illumination variation.

According to the analysis in the previous sections, it is difficult to accurately segment the semiconductor shielding layer region from these images by traditional edge detection methods. It can be seen from Figure 13, relatively speaking, hat the method based on the deep neural network is well adapted to the light change and cutting mark interference, and the semiconductor shielding layer region is successfully segmented.

It can be seen from the fourth column of Figure 13 that a small part of the outer edge region of the insulation layer is retained in the segmentation results of the standard UNet model, but this part of the region is correctly predicted by the proposed method.

As seen from the last two columns in Figure 13, the standard UNet model incorrectly predicts the area of the ground insulation layer near the semiconductor shielding layer as the semiconductor shielding layer. If such a prediction is used for measurements, there will be a serious error. The proposed method obtained relatively correct segmentation results.

From the edge details of the segmentation results, such as the third column and the fifth column, the internal and external edges in the segmentation results of the comparison method are relatively rough, while the segmentation results of the proposed method are closer to the actual edge situation.

Therefore, although both methods successfully segment the semiconductive layer area, the proposed method performs better in detail.

## 6. Conclusions

In this paper, a semiconductive shielding layer segmentation method based on the convolutional neural network is proposed, and it is a typical application for polymer materials. The main novelties of this study are as follows. First, the inception structure is introduced to make the network more robust to scale. Second, the residual connection is employed to improve the U-shaped structure. Third, a weighted loss function is proposed especially for this task to force the network to pay more attention to the pixels that are difficult to classify. Finally, the prediction refinement step based on prior knowledge is proposed to refine the network prediction results. The experimental results demonstrate that the proposed method can deal with the task of semiconductive shielding layer segmentation. In the future, we intend to improve our method based on other architectures, such as DenseNet [21], richer features [22], and CRF-RNN [23].

## Figures and Tables

**Table 1:** Prediction performance comparisons using different stages.

Model	IoU	Dice	Precision
3 stages	96.02	96.29	97.35
4 stages	96.63	96.62	97.71
5 stages	96.89	96.88	97.83

**Table 2:** Prediction performance comparisons of using different stages. S1, Submodel 1.

Model	IoU	Dice	Precision
S1	93.40	93.11	94.08
S2	96.29	96.27	97.26
S3	96.89	96.88	97.83

## Abbreviations

The following abbreviations are used in this manuscript:

CNNs	Convolutional neural networks
FCNs	Fully convolutional networks
BN	Batch normalization
ReLU	Rectified linear unit
BCE	Binary cross-entropy loss
IoU	Intersection over union
FP	False positive
TP	True positive
FN	False negative

## Appendix A

**Table A1:** Architecture of the contracting path.

Stage Index	Block Name	Output Size
	Network input	224 × 224 × 1
Stage 1	Incep-block 1-1 Incep-block 1-2 Residual Connection Max-pooling	224 × 224 × 32 224 × 224 × 32 224 × 224 × 32 112 × 112 × 32
Stage 2	Incep-block 2-1 Incep-block 2-2 Residual Connection Max-pooling	112 × 112 × 64 112 × 112 × 64 112 × 112 × 64 56 × 56 × 64
Stage 3	Incep-block 3-1 Incep-block 3-2 Residual Connection Max-pooling	56 × 56 × 128 56 × 56 × 128 56 × 56 × 128 28 × 28 × 128
Stage 4	Incep-block 4-1 Incep-block 4-2 Residual Connection Max-pooling	28 × 28 × 256 28 × 28 × 256 28 × 28 × 256 14 × 14 × 256
Stage 5	Incep-block 5-1 Incep-block 5-2 Residual Connection	14 × 14 × 512 14 × 14 × 512 14 × 14 × 512

**Table A2:** Architecture of the expansive path.

Stage Index	Block Name	Output Size
Stage 4	Deconvolution Concatenation Incep-block 6-1 Residual Connection Incep-block 6-2 Residual Connection	28 × 28 × 256 28 × 28 × 512 28 × 28 × 256 28 × 28 × 256 28 × 28 × 256 28 × 28 × 256
Stage 3	Deconvolution Concatenation Incep-block 7-1 Residual Connection Incep-block 7-2 Residual Connection	56 × 56 × 128 56 × 56 × 256 56 × 56 × 128 56 × 56 × 128 56 × 56 × 128 56 × 56 × 128
Stage 2	Deconvolution Concatenation Incep-block 8-1 Residual Connection Incep-block 8-2 Residual Connection	112 × 112 × 64 112 × 112 × 128 112 × 112 × 64 112 × 112 × 64 112 × 112 × 64 112 × 112 × 64
Stage 1	Deconvolution Concatenation Incep-block 9-1 Residual Connection Incep-block 9-2 Residual Connection	224 × 224 × 32 224 × 224 × 64 224 × 224 × 32 224 × 224 × 32 224 × 224 × 32 224 × 224 × 32
	Network output	224 × 224 × 1
